# Response to Correspondence: Polylactic Acid: Is It Everything the Same?

**DOI:** 10.1111/jocd.16725

**Published:** 2024-12-12

**Authors:** Airá Novello Vilar, Vitoria Azulay, Estevão Vargas, Mayra Ianhez, Regina Casz Schechtman

**Affiliations:** ^1^ Institute of Dermatology Professor Rubem David Azulay Santa Casa de Misericórdia Do Rio de Janeiro Rio de Janeiro Brazil; ^2^ Universidade Federal de Goiás Goiânia Brazil

**Keywords:** collagen, histopathology, polylactic acid

Dear Authors,

We appreciate your time and effort dedicated to providing feedback on our manuscript. The primary objective of our study is explicitly stated in the following sentence: “The objective of this study is to microscopically analyze the composition of pure products, in vitro, 1 day after dilution of two existing PLLA formulations, Sculptra and Elleva [1, 2].”

As such, it is beyond the scope of this publication or our research focus to perform cell culture analysis, as this does not necessarily correlate with in vivo tissue effects. Similarly, patient follow‐up was not the subject of this letter to the editor.

The significance of this manuscript lies in its demonstration of previously undocumented microscopic differences between the two PLLA formulations examined. To further clarify these differences and in response to the scientific relevance of the issue, we have included histopathological analysis of nodules from Sculptra and Elleva (Figures 1A,B and 2A,B). Notably, we observed crystals within multinucleated giant cells in the Sculptra sample, which were absent in the Elleva sample. Additionally, Alcian blue staining revealed the presence of acidic mucins within the cytoplasm of multinucleated giant cells in the Elleva nodule, a feature not seen in the Sculptra sample. This histopathological analysis supports the previously observed in vitro findings and reinforces the clinical and tissue‐reaction relevance, particularly regarding complications such as nodule formation.

**FIGURE 1:**
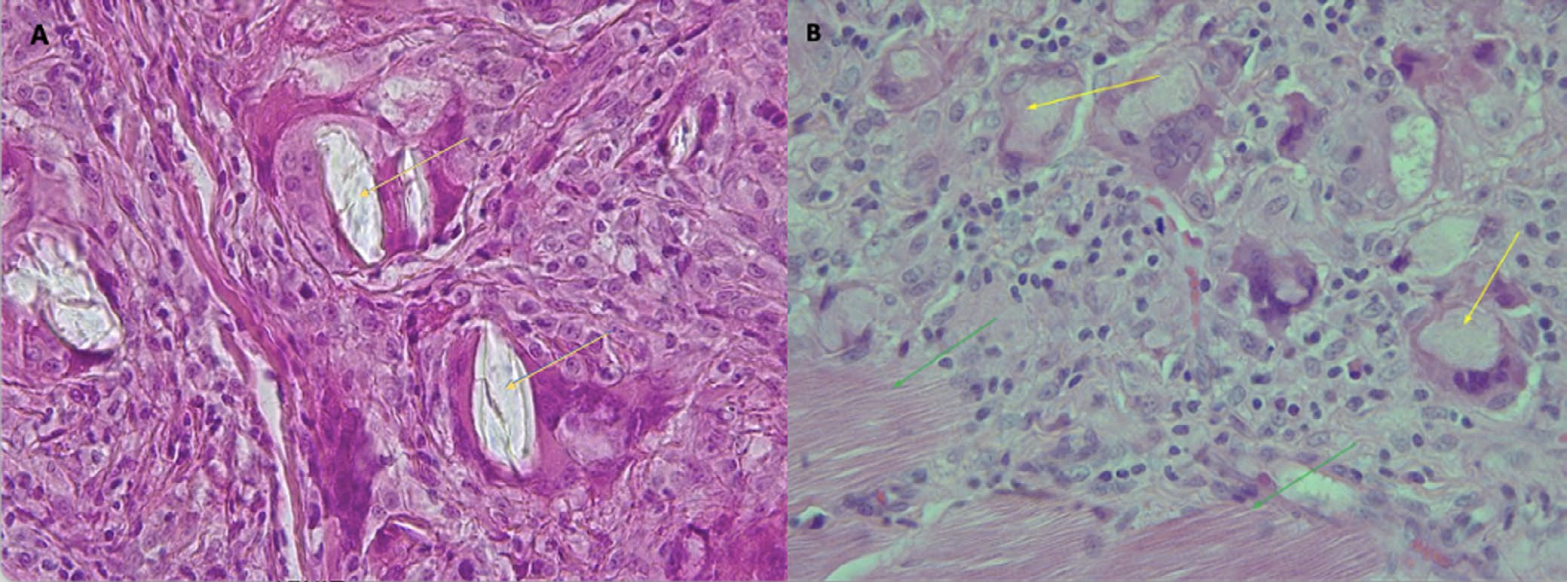
(A) Sculptra: Hematoxylin and eosin staining (HE), 400×, showing refringent crystals (yellow arrow) inside multinucleated foreign body giant cells. (B) Elleva: Hematoxylin and eosin staining (HE), 400×, showing granulomatous reaction and eosinophilic amorphous material (yellow arrow) inside multinucleated foreign body giant cells and absence of crystals. We also draw attention to the proximity of the material to muscle tissue (green arrow).

**FIGURE 2:**
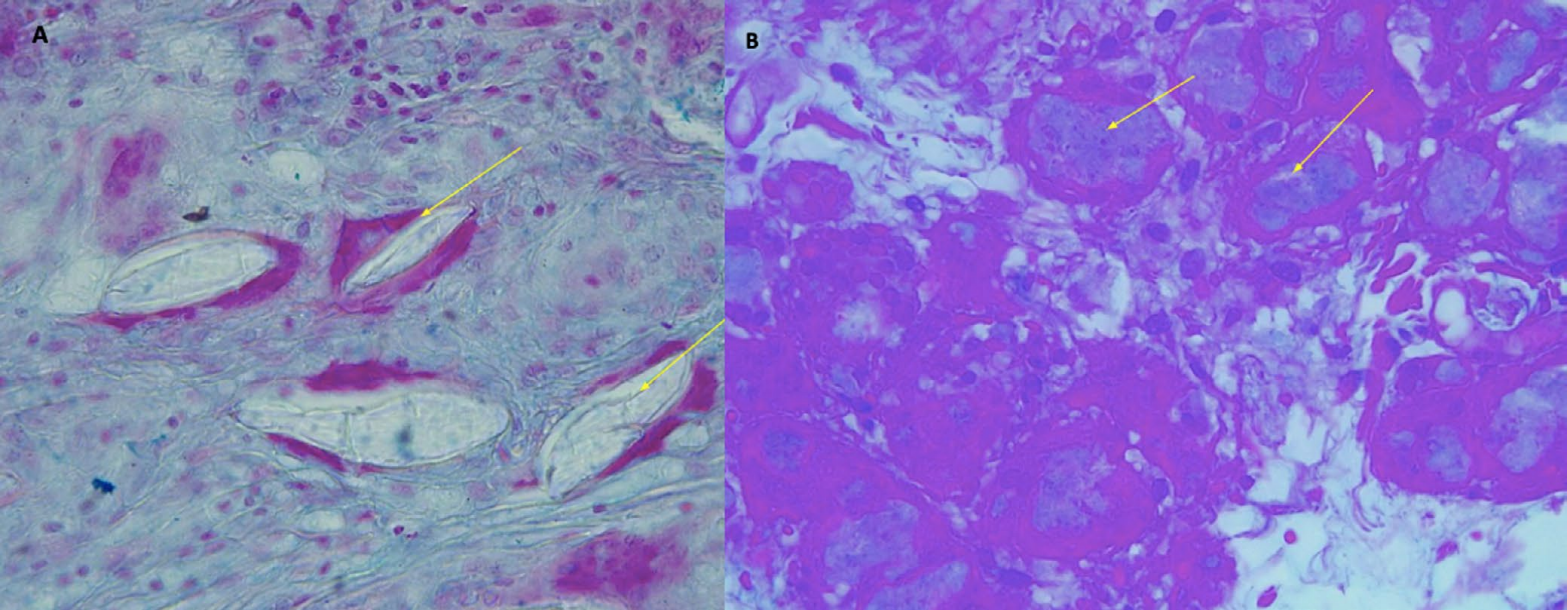
(A) Sculptra: Alcian blue staining, 400×, showing refringent crystals (arrows) inside the foreign body multinucleated giant cells (granulomatous reaction) and negative Alcian blue staining. (B) Elleva: Alcian blue staining, 400×, demonstrating positive acidic material inside multinucleated giant cells and absence of crystals, even with polarization.

All references are based on previously published manuscripts that contribute to the current literature on the subject. These citations are accurately integrated into the text, offering readers further resources for in‐depth exploration. For instance, the referenced ultrasound findings are left for readers to interpret critically, as it was not our intention to draw definitive conclusions in this communication [3, 4, 5, 6].

The insights provided by the authors of the correspondence are undeniably valuable, yet they should be explored in future studies to substantiate their opinions and validate their relevance.

We appreciate the opportunity to submit our response and would like to emphasize that this study was conducted without any financial support from industry, including material costs. There are no conflicts of interest related to this manuscript.

In conclusion, as clearly articulated, the aim of this study aligns perfectly with the title of the article and underscores the pertinent microscopic differences between the PLLA products analyzed—a finding we reaffirm in this response through in vivo correlations [7].

## Author Contributions

A.N.V. performed the research, designed the research study, and wrote the paper. V.A. performed the research, designed the research study, and wrote the paper. E.V. wrote the paper and analyzed the data. M.I. wrote the paper and analyzed the data. R.C.S. contributed essential tools and analyzed the data. All authors have read and approved the final version of the manuscript.

## Ethics Statement

The study was approved by the ethical review board.

## Conflicts of Interest

The authors declare no conflicts of interest.

## Data Availability

All data generated or analyzed during this study are included in this article. Further enquiries can be directed to the corresponding author.

## References

[1] A. N. Vilar , V. Azulay , E. Vargas , and R. C. Schechtman , “Polylactic Acid: Is It Everything the Same?,” Journal of Cosmetic Dermatology (2024): Epub ahead of print, 10.1111/jocd.16539. PMC11626334 39161304

[2] J. E. Chicarelli Martin , “Commentary to ‘Polylactic Acid: Is It Everything the Same? [Sic]’,” Journal of Cosmetic Dermatology (2024): Epub ahead of print, 10.1111/jocd.16603. PMC11743288 39324645

[3] R. Fitzgerald , L. M. Bass , D. J. Goldberg , M. H. Graivier , and Z. P. Lorenc , “Physiochemical Characteristics of Poly‐L‐Lactic Acid (PLLA),” Aesthetic Surgery Journal 38, no. suppl_1 (2018): S13–S17, 10.1093/asj/sjy012. 29897517

[4] M. D. Palm and M. P. Goldman , “Patient Satisfaction and Duration of Effect With PLLA: A Review of the Literature,” Journal of Drugs in Dermatology 8, no. 10 Suppl (2009): s15–s20. 19891117

[5] D. Vleggaar , “Facial Volumetric Correction With Injectable Poly‐L‐Lactic Acid,” Dermatologic Surgery 31, no. 11 Pt 2 (2005): 1511–1517, discussion 1517–1518. 16416633 10.2310/6350.2005.31236

[6] M. Ianhez , E. de Goés , G. Silva Freire , et al., “Complications of Collagen Biostimulators in Brazil: Description of Products, Treatments, and Evolution of 55 Cases,” Journal of Cosmetic Dermatology 23 (2024): 2829–2835, 10.1111/jocd.16343. 38693639

[7] R. M. Sigrist , M. G. O. de Noronha , S. S. Borelli , S. P. Teixeira , H. L. X. Funes , and L. M. Lourenço , “Dynamic Ultrasound Evaluation of Body Fillers and Biostimulators in the Buttocks of Fresh‐Frozen Specimen,” Journal of Cosmetic Dermatology 21, no. 11 (2022): 5621–5627, 10.1111/jocd.15333. 36029286

